# Differential Microbial Diversity in* Drosophila melanogaster*: Are Fruit Flies Potential Vectors of Opportunistic Pathogens?

**DOI:** 10.1155/2017/8526385

**Published:** 2017-11-06

**Authors:** Luis A. Ramírez-Camejo, Génesis Maldonado-Morales, Paul Bayman

**Affiliations:** ^1^Centro de Biodiversidad y Descubrimiento de Drogas, Instituto de Investigaciones Científicas y Servicios de Alta Tecnología (INDICASAT AIP), Edificio 219, Ciudad del Saber, Apartado 0843-01103, Ciudad de Panamá, Panama; ^2^Department of Biology, University of Puerto Rico, Río Piedras, PR, USA

## Abstract

*Drosophila melanogaster* has become a model system to study interactions between innate immunity and microbial pathogens, yet many aspects regarding its microbial community and interactions with pathogens remain unclear. In this study wild* D. melanogaster* were collected from tropical fruits in Puerto Rico to test how the microbiota is distributed and to compare the culturable diversity of fungi and bacteria. Additionally, we investigated whether flies are potential vectors of human and plant pathogens. Eighteen species of fungi and twelve species of bacteria were isolated from wild flies. The most abundant microorganisms identified were the yeast* Candida inconspicua* and the bacterium* Klebsiella* sp. The yeast* Issatchenkia hanoiensis* was significantly more common internally than externally in flies. Species richness was higher in fungi than in bacteria, but diversity was lower in fungi than in bacteria. The microbial composition of flies was similar internally and externally. We identified a variety of opportunistic human and plant pathogens in flies such as* Alcaligenes faecalis*,* Aspergillus flavus*,* A. fumigatus*,* A. niger*,* Fusarium equiseti/oxysporum, Geotrichum candidum*,* Klebsiella oxytoca*,* Microbacterium oxydans*, and* Stenotrophomonas maltophilia*. Despite its utility as a model system,* D. melanogaster* can be a vector of microorganisms that represent a potential risk to plant and public health.

## 1. Introduction

The microbiota of wild* Drosophila melanogaster* is distinct from that of flies from laboratory stocks [1–4]. A wide range of bacteria from Proteobacteria, Firmicutes, and Bacteroidetes phyla, among others, have been reported from* Drosophila* [2, 4]. In contrast, fungi are poorly characterized in* Drosophila*, with most studies focusing on taxonomy, ecology of yeast in the gut, and importance in the diet [5–7].

In the early 20th century, some* Drosophila* species were considered a potential vector of disease because its frequency near excrement and public toilets [8]. In a recent study, the Mediterranean fruit flies* Ceratitis capitata* and* D. melanogaster* were shown to transmit* Escherichia coli* to intact apple fruits, suggesting they are potential vectors of pathogens [9, 10]. This is a disturbing conclusion because* D. melanogaster* has a worldwide distribution and visits a wide variety of human foods [11]. Hence, fruit flies have been considered a common pest in the food industry [12]. In one instance, discovery of a population of fruit flies in an operating room at a New Jersey hospital resulted in the disruption of a dozen surgeries [13].

By the start of the 21st century,* Drosophila* had been established as a model system for immune studies after analysis of its genome revealed unsuspected sophistication and similarity to the mammalian innate immune system [14–16]. The use of* Drosophila* for studies of virulence and pathogen interactions requires a deeper knowledge of its microbial symbionts and their internal and external distributions.

In this study, we isolated microorganisms from wild* D. melanogaster* to answer the following questions: (1) how is the microbiota of* Drosophila* distributed spatially? We hypothesized that some microorganisms are found in both external and internal tissues of flies [17]; however, other bacteria and fungi will be limited to either internal or external surfaces. (2) Are culturable fungi and bacteria equally diverse in flies? We hypothesize that bacteria will be more diverse than fungi in* D. melanogaster* because they form stable relationships with flies in nature and are important food sources for larvae [4]. (3) Are fruit flies potential vectors of opportunistic pathogens? Because fruit flies can transport microorganisms of human concern [9, 10, 18], we hypothesize that some fungi and bacteria isolated from wild flies will be potential vectors of plant and animal pathogens.

## 2. Methodology

### 2.1. Sampling, Culture Media, and Isolation of Microorganisms

Wild females of* Drosophila melanogaster* were collected in Puerto Rico from tropical fruits (mango, orange, starfruit, and jackfruit). Only culturable microorganisms were included in order to obtain data and isolates for a related project on probiotics [19]. Flies were attracted using glass jars with fruit and jars were covered with gauze after enough flies entered the jar. This was repeated four times at one-month intervals for a total of 160 flies.

Fungi were isolated on Potato Dextrose Agar (PDA) and bacteria on Tryptic Soy Agar (TSA); they are common and nonselective media that provide enough nutrients to encourage growth of a range of fungi and bacteria, respectively. Fungi and bacteria were isolated externally and internally from 40 flies per sample as follows: 5 flies each were placed on plates of PDA and TSA and allowed to walk on the surface for five minutes. Another 10 flies per sample were then anesthetized with CO_2_, placed in a microcentrifuge tube with sterile water and Tween 80 (0.01%) and mixed in a vortex for 1 minute to release microbial cells from body surfaces [20]. The wash solution was then streaked on the culture media described above. Another 10 flies were surface-sterilized in 70% ethanol for 1 min, rinsed three times with sterile water, and placed on PDA and TSA, five per plate. The guts of another 10 flies surface-sterilized were extracted using a sterile forceps and needle. Guts were rinsed in sterile water and streaked with a sterile loop on PDA and TSA (5 guts per plate). Plates were incubated at 28°C for seven days to allow microbial growth.

Microorganisms were isolated every day and plated in a 2 mL glass vial with PDA or TSA. Microorganisms were grouped by morphospecies based on morphological characteristics, for example, colony size, color, texture, and type of margin.

### 2.2. DNA Extraction

One fungal isolate from each morphospecies was cultured in Potato Dextrose Broth (PDB), filtered, and macerated in liquid nitrogen. DNA was extracted using a phenol-chloroform method [21]. The same procedure was used for* Drosophila*. One bacterial isolate from each morphospecies was cultured in liquid Nutrient broth for 24–48 hours, cells were then lysed by heat-shock and suspended in 1 mL of sterile distilled water, and DNA was diluted to 4–30 ng/*μ*L for PCR.

### 2.3. Polymerase Chain Reaction and Sequencing

For fungi, the nuclear ribosomal Internal Transcribed Spacer (ITS) was amplified using primers ITS 1F and ITS 4 [21, 22]. For bacteria, part of the 16S ribosomal gene was amplified using primers fD1 and rP2 [23]. For* Drosophila*, the mitochondrial cytochrome oxidase subunit I was amplified with primers LCO1490 and HCO2198 [24]. Amplicons were 600–1300 nucleotides for bacteria, 200–500 nucleotides for fungi, and ~600 nucleotides for* Drosophila*. PCR products were cleaned using Exo-Sap (Fermentas) and sequenced in the Sequencing and Genotyping Facility (SGF) at UPR-RP. Sequences from flies, bacteria, and fungi (GenBank accession numbers KU238836–KU238862) were corrected with Sequencher software and identified by BLASTn searches in GenBank. Names assigned were based on >98% identity (Table 1).

### 2.4. Statistical Analysis

EstimateS (version 9.1.0 for Mac) was used to compare the richness (Chao 1), diversity (Shannon index), and composition (Bray-Curtis index) of communities in flies (http://viceroy.eeb.uconn.edu/estimates/). Species accumulation curves were obtained using the variable *S* (est). Chi square (*χ*^2^) tests were used to compare differences between external versus internal microbial communities.

## 3. Results

### 3.1. Distribution and Diversity of Microorganisms Isolated from* Drosophila melanogaster*

We isolated 314 microorganisms from wild* Drosophila melanogaster*, including 171 fungi and 143 bacteria, which were grouped into 18 and 12 morphospecies, respectively (Table 1). The most abundant fungus identified was the yeast* Candida inconspicua* which represented 49% of fungi isolated. The most common bacterial genus was* Klebsiella* (22%).

**Figure 1:**
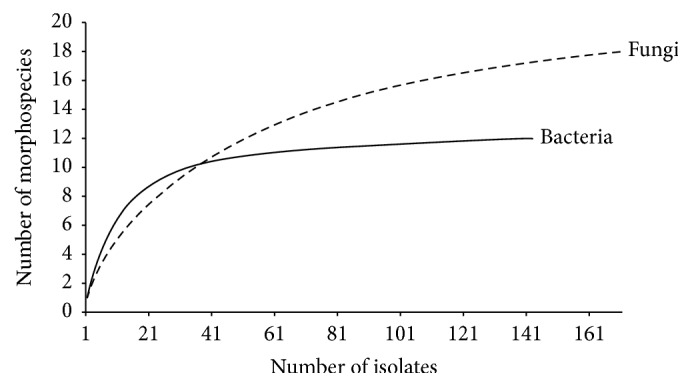
Species accumulation curves for fungal and bacterial morphospecies isolated from female* Drosophila melanogaster* in Puerto Rico. Species order was randomized 100 times. Fungal and bacterial species are represented with dashed and solid lines, respectively.

Species richness estimated as Chao 1 was 20 and 12, in fungal and bacterial communities, respectively. Species accumulation curves showed that for bacteria sampling was sufficient, assuming that our morphospecies did not contain cryptic species. For fungi the sampling was insufficient (Figure 1). The microbial diversity estimated with Shannon's index (*H*′) was 1.87 for fungi and 2.23 for bacteria.

Only the yeast* Issatchenkia hanoiensis* (*χ*^2^ = 6.2, *P* < 0.013) was significantly more common in fly guts than external surfaces (Table 1). The remaining microorganisms did not differ significantly in frequency between external and internal origin (*P* > 0.05).

The species composition did not differ significantly between internal and external microbiotas, either for fungi (Bray-Curtis = 0.68) or for bacteria (Bray-Curtis = 0.71).

### 3.2. Potential Opportunistic Pathogens Isolated from* Drosophila melanogaster*

Bacteria and fungi isolated from* Drosophila melanogaster* included opportunistic pathogens of humans and animals, including* Klebsiella oxytoca*,* Alcaligenes faecalis*,* Microbacterium oxydans*,* Stenotrophomonas maltophilia*,* Aspergillus fumigatus*,* A. flavus*, and* A. niger* (Table 1). Also,* A. flavus*,* A. niger*,* Fusarium equiseti/oxysporum*, and* Geotrichum candidum* are considered opportunistic plant pathogens (Table 1).

## 4. Discussion

### 4.1. Differences between Bacteria and Fungi Isolated from* Drosophila melanogaster*

This study was limited to culturable microorganisms which were used for experiments on probiotics [19]. However, our protocol excluded the majority of bacteria and many fungi which are nonculturable or require specialized media or culture conditions [25].

The richness of fungal morphospecies was higher than that of bacteria (Figure 1). The accumulation curves for flies levelled off, suggesting that nearly all the culturable bacterial species present in flies were detected, but not for fungi. These results contradict a previous study where the fungal communities associated with different* Drosophila* species sampled around the world were less rich that those of bacteria [26]. However, that study only focused on yeasts isolated from guts of flies, which constitute the vast majority of known* Drosophila*-associated fungi.

In contrast, even though the fungal community is richer in species, the bacteria community is more diverse in* D. melanogaster* (*H*′, fungi = 1.87 versus bacteria = 2.23). This suggests that the population sizes of different bacterial species in the flies are more equitable. This is supported by two studies in which bacterial diversity exceeds fungal diversity in* Drosophila* populations [2, 26].

The yeast* Issatchenkia hanoiensis* was more abundant in internal parts of flies than externally (*P* < 0.013). Yeasts are common* Drosophila* symbionts, and some are food sources for* Drosophila* [26]. Yeast like* Saccharomyces cerevisiae* can survive passage through the digestive tract of flies because the constituents of spore walls are more resistant than vegetative cells [27]. It would be interesting to examine if* I. hanoiensis* provides any benefit to flies, for example, food source for larvae, roles in attraction, ovoposition, development, or protection against pathogens [5, 7, 28–31].* I*.* hanoiensis* was first described in 2003 from insect frass; it has not previously been reported from* Drosophila* [32].

Apart from* Issatchenkia*, species composition internally versus externally in flies was similar for fungi (Bray-Curtis = 0.68) and for bacteria (Bray-Curtis = 0.71). This result contrasts with a previous microbiome study where the internal bacterial communities were a reduced subset of the external bacterial communities, suggesting that flies can control the microorganisms in the digestive tract and internal tissues [3].

### 4.2. *Drosophila melanogaster* as a Potential Vector of Pathogens

*Drosophila melanogaster* can carry opportunistic pathogens of humans [18]. We isolated the Gram-negative bacterium* Klebsiella oxytoca* which has been reported as a causal agent of hemorrhagic colitis, and* Alcaligenes faecalis* was previously associated with infections in newborns [33, 34]. Other microorganisms isolated in this study were also reported as emerging clinical pathogens, for example,* Microbacterium oxydans* and* Stenotrophomonas maltophilia* [35–38]. We also isolated three opportunistic pathogens capable of causing animal and human aspergillosis:* Aspergillus fumigatus*,* A*.* flavus*, and* A. niger* [39–42]. Their presence is not surprising because they are ubiquitous in nature with abundant airborne conidia [43].

Fruit flies as sources of contamination could represent a public health risk, especially to patients with compromised immune systems. For example, Mediterranean fruit flies* (Ceratitis capitata)* exposed to fecal material enriched with GFP-tagged* Escherichia coli* are capable of transmitting* E. coli* to intact apples in a cage model system [10]. The same was seen in* D. melanogaster* [9].

In addition, plant pathogens of agricultural concern were documented in the sampled flies, for example,* A. niger, Fusarium equiseti/oxysporum,* and* Geotrichum candidum* [44–47].* A. flavus* causes substantial problems in agriculture as a source of aflatoxins and frequently enters plants through insect-induced wounds [40, 48].

Almost one hundred years ago,* D. melanogaster*, commonly found in exposed fruit in grocery stores and houses, was reported as “not an efficient disease carrier” [8]. This was based on the fact that* D. melanogaster* is rarely attracted to excrement. However, the studies mentioned support our hypothesis that flies might serve as vectors for opportunistic pathogens to humans and plants. More experiments are necessary to clarify the identity and virulence of the opportunistic pathogens found in this study.

## 5. Conclusions

The isolation of culturable microorganisms from wild* D. melanogaster* suggests that its microbiota is rich, diverse, and distributed throughout internal and external surfaces.* Issatchenkia hanoiensis* was identified as common component of the fly microbiota. Other microorganisms are related to opportunistic human pathogens, which may represent a public health risk, indicating* D. melanogaster* is a potential vector of disease.

## Figures and Tables

**Table 1:** Identification and distribution of fungi and bacteria on and in fruit flies. GenBank accession numbers are for ITS sequences of fungi and 16S rDNA sequences for bacteria. The last six columns show numbers of each microorganism isolated with the following protocols: flies that walked across petri plates; flies washed in 0.01% Tween 80; surface-sterilized flies; and guts removed from flies.

Strain	Species	GenBank accession ID	Highest hit	GenBank accession ID	% identity	External isolation		Internal isolation		Total	*P* value
						Walked	Washed	Insect	Gut		
	Fungus										
H137	*Candida inconspicua*	KU238836	*Candida inconspicua*	KT207004	100	27	20	23	14	84	0.275
H35	*Penicillium crustosum/commune*	KU238837	*Penicillium commune*	KR012904	100	10	5	6	7	28	0.705
H127	*Issatchenkia hanoiensis*	KU238838	*Issatchenkia hanoiensis*	FJ153178	99	1	1	9	2	13	0.013^*∗*^
H66	*Aspergillus versicolor/sydowii/nidulans*	KU238839	*Aspergillus sydowii*	KT989398	100	3	1	0	4	8	1.000
H11	*Aspergillus fumigatus*	KU238840	*Aspergillus fumigatus*	AB298709	100	2	1	2	0	5	0.655
H37	*Fusarium *sp.	KU238841	*Fusarium equiseti*	HQ332532	100	1	1	2	1	5	0.655
H44	*Galactomyces candidum/geotrichium*	KU238842	*Galactomyces candidum*	KJ579946	99	1	1	1	1	4	1.000
H102	*Pichia membranifaciens*	KU238843	*Pichia membranifaciens*	FJ231462	99	3	0	1	0	4	0.317
H7	*Rhizopus *sp.	—	—	—	—	0	3	0	0	3	0.083
H24	*Fusarium equiseti/oxysporum*	KU238844	*Fusarium equiseti*	KJ174399	100	2	0	0	1	3	0.564
H77	*Penicillium citrinum/griseofulvum*	KU238845	*Penicillium citrinum*	KU681430	98	0	2	1	0	3	0.564
H31	*Aspergillus niger*	KU238846	*Aspergillus niger*	KP748369	99	1	1	1	0	3	0.564
H46	*Geotrichum candidum*	KU238847	*Geotrichum candidum*	KF713518	100	1	1	0	0	2	0.157
H69	*Aspergillus flavus/oryzae*	KU238848	*Aspergillus flavus*	KU360621	100	0	1	0	0	1	0.317
H70	*Aspergillus niger/tubingensis*	KU238849	*Aspergillus niger*	EU645723	98	0	0	1	0	1	0.317

	Bacteria										
B82	*Klebsiella *sp.	KU238850	*Klebsiella sp.*	FN178363	99	5	6	8	13	32	0.077
B5	*Bacillus *sp.	KU238851	*Bacillus pumilus*	KU517819	99	7	5	4	6	22	0.670
B30	*Klebsiella oxytoca*	KU238852	*Klebsiella michiganensis*	KP717391	99	6	6	3	5	20	0.371
B44	*Klebsiella pneumoniae/variicola*	KU238853	*Klebsiella variicola*	KT895843	99	3	1	4	6	14	0.109
B22	*Erwinia *sp.	KU238854	*Uncultured Erwinia *sp.	HE575588	99	3	4	3	1	11	0.366
B105	*Bacillus pumilus/safensis*	KU238855	*Bacillus pumilus*	KU239978	99	2	0	3	4	9	0.096
B39	*Stenotrophomonas maltophilia*	KU238856	*Stenotrophomonas maltophilia*	LT222226	99	2	2	3	2	9	0.739
B84	*Micrococcus luteus/yunnanensis*	KU238857	*Micrococcus luteus*	KT901825	100	4	0	4	0	8	1.000
B43	*Alcaligenes faecalis*	KU238858	*Alcaligenes faecalis*	KU179370	99	2	1	1	2	6	1.000
B8	*Microbacterium oxydans*	KU238859	*Microbacterium oxydans*	KT580637	99	0	1	0	0	1	0.317

	Drosophila										
D1	*Drosophila melanogaster*	KU238860	*Drosophila melanogaster*	KP161877	99						
D2	*Drosophila melanogaster*	KU238861	*Drosophila melanogaster*	KP161877	99						
D3	*Drosophila melanogaster*	KU238862	*Drosophila melanogaster*	KP161877	99						

Asterisk represents significant differences by Chi square test.
